# Supplementary material for: Serum proteomics reveals high-affinity and convergent antibodies by tracking SARS-CoV-2 hybrid immunity to emerging variants of concern
Source: Front Immunol. 2025 Feb 25;16:1509888. doi: 10.3389/fimmu.2025.1509888 (PMC11893383; doi:10.3389/fimmu.2025.1509888)
Supplement: Supplementary file 1 [file DataSheet1.pdf]

Supplementary Material:

Serum proteomics reveals high-affinity and convergent antibodies by tracking SARS-CoV-2 hybrid immunity to emerging variants of concern

1 SUPPLEMENTARY DATA

2 SUPPLEMENTARY TABLES AND FIGURES

2.1 Tables

2.2 Figures

<table>
<tr><th>donor sequence id</th><th>public sequence id</th><th>mAb</th><th>donor</th><th>observed affinity</th><th>% HC match</th><th>% CDR-H3 match</th></tr>
<tr><td>cluster__20832-1__AB5-spike__size__1</td><td>BD56-1725</td><td></td><td>AB5</td><td>RBD</td><td>87.9</td><td>82.4</td></tr>
<tr><td colspan="7">QVHLVQSGAEVKKPGSSVKVSCKASGGTFSSYTISWVRQAPGQGLEWMGRIIPFLGVANDAQKFQGRVTITADKSTSTAYLELGSLTSEDTAVYYC AKDQGYSGSGSNRYFDL WGRGTLVSVSS
||.|||||||||||||||||||.||||||||||.|||||||||||||||||||.||.||.||.|||||||||||||||||.||.||.|||||.||| |.|.||||||||.|||| |||||||.|||
QVQLVQSGAEVKKPGSSVKVSCEASGGTFSSYTFSWVRQAPGQGLEWMGRIIPILGIANYAQNFQGRVTITADKSTSTAYVELSSLRSEDTAIYYC ARDIGYSGSGSNWYFDL WGRGTLVTVSS</td></tr>
<tr><td>cluster__44992-0__AB5-spike__size__1</td><td>C690</td><td>AB5-20</td><td>AB5</td><td>RBD</td><td>87.4</td><td>80.0</td></tr>
<tr><td colspan="7">QVQLVESGGGVVQPGRSLRLSCTASGFTFRHYGLHWVRQAPGKGLEWVAVILYDGSDEDYRDSVKGRFTISRDNSKNTLYLQMNSLRPEDTAVYYC AKQSGPYCSGGNCYSGHFDY WGQGTPVSVSS
||||||||||||||||||||||.|||||||.||.|||||||||||||||||.||||...|.|||.||||||||||||||||||||||.|||||||| |||.|||||||||..|.||| |||||.|.|||
QVQLVESGGGVVQPGRSLRLSCAASGFTFRTYGMHWVRQAPGKGLEWVAVISYDGSKKYYGDSVRGRFTISRDNSKNTLYLQMNSLRAEDTAVYYC AKQGGPYCSGGNCNVGYFDY WGQGTLVTVSS</td></tr>
<tr><td>cluster__2336-0__AB6-spike__size__1</td><td>pT1611</td><td>AB6-10</td><td>AB6</td><td>RBD</td><td>92.3</td><td>90.9</td></tr>
<tr><td colspan="7">EVQLVESGGGLVQPGGSLRLSCAASGVIVSSNYMSWVRQAPGEGLEWVSIMYAGGSTFYADSVKGRFTISRDNSKNTLFLQMNSLRPEDTAVYYC VRDLQDFGMDV WGQGTTVTVSS
|||||||||||.|||||||||||||||.||.|||||||||||.|||||||.|||||||||||||||||.|||||||||.|||||||.|||||||| ||||||.|||| |||||||||||
EVQLVESGGGLIQPGGSLRLSCAASGVTVSRNYMSWVRQAPGKGLEWVSIIYAGGSTFYADSVKGRFTVSRDNSKNTLYLQMNSLRAEDTAVYYC VRDLQDYGMDV WGQGTTVTVSS</td></tr>
<tr><td>cluster__31008-0__AB5-spike__size__1</td><td>R40-1C8</td><td>AB5-13</td><td>AB5</td><td>SPIKE</td><td>90.6</td><td>81.8</td></tr>
<tr><td colspan="7">EVQLVESGGGLVQPGGSVRLTCAASGVTVSSNYMSWVRQAPGKGLEWVSLLYAGGSTEYADSVKGRFTISRHNSKNTLFLQMNNLRPEDTAVYYC VRDLQFYGMDV WGQGTTVTVSS
|||||||||||||||||.||.|||||||||.||||||||||||||||||..||||||.||||||||||||||||||||.||||.||.|||||||| ||||..||||| |||||||||||
EVQLVESGGGLVQPGGSLRLSCAASGVTVSRNYMSWVRQAPGKGLEWVSVIYAGGSTFYADSVKGRFTISRHNSKNTLYLQMNSLRVEDTAVYYC VRDLVDYGMDV WGQGTTVTVSS</td></tr>
<tr><td>cluster__15916-0__AB5-spike__size__1</td><td>BD56-411</td><td>AB5-18</td><td>AB5</td><td>RBD & SPIKE</td><td>90.6</td><td>83.3</td></tr>
<tr><td colspan="7">DVQLVESGGGLVQPGGSLRLSCAASGVTVSSNYMNWVRQAPGEGLQWVSIMYSGGSTFYADSVKGRFTISRDSSKNTLFLQMNSLRIEDTAVYYC ARDLQEAGGMDV WGQGTTVTVSS
.|||||||||||.|||||||||||||||||||||||||||||.||.|||..||||||.||||||||||||||.|||||.|||||||.|||||||| ||||.|.||||| |||||||||||
EVQLVESGGGLVRPGGSLRLSCAASGVTVSSNYMNWVRQAPGKGLEWVSLFYSGGSTYYADSVKGRFTISRDNSKNTLYLQMNSLRDEDTAVYYC ARDLREVGGMDV WGQGTTVTVSS</td></tr>
<tr><td>cluster__17473-0__AB5-spike__size__1</td><td>368.01a.A.0031</td><td></td><td>AB5</td><td>RBD</td><td>93.2</td><td>91.7</td></tr>
<tr><td colspan="7">EVQLVESGGGLVQPGGSLRLSCAASGVTVSSNYMSWVRQAPGKGLQWISLIYAGGSTFYADSVKGRFTISRDNSKNTLYLQMNRLRVEDTAVYYC ARDLDVVGGMDV WGQGTTVTVSS
|||||||||||.||||||||||||||.||||||||||||||||||.|.||||.||||||||||||||||||||||||||||||.||.|||||||| ||||||.||||| |||||||||||
EVQLVESGGGLIQPGGSLRLSCAASGITVSSNYMSWVRQAPGKGLEWVSLIYSGGSTFYADSVKGRFTISRDNSKNTLYLQMNSLRAEDTAVYYC ARDLDVRGGMDV WGQGTTVTVSS</td></tr>
<tr><td>cluster__14976-0__AB6-spike__size__1</td><td>CQT218</td><td></td><td>AB6</td><td>RBD</td><td>87.3</td><td>84.6</td></tr>
<tr><td colspan="7">EEHLVESGGGLVQPGGSLRLSCAASGFMFTNFWMTWVRQAPGKGLEWVANIQQDGSEKYYLDSVKGRFTISRDNTKNSVYLEMISLRAEDTAVYYCARDLGVLWFGED-YSGQGTLVTVSS
|..||||||||||||||||||||||||.|...||.|||||||||||||.||.||||||||.|||||||||||||.|||.||.|.||||||||||||||||||||||.|.|.||||||||||
EVQLVESGGGLVQPGGSLRLSCAASGFSFSSYWMSWVRQAPGKGLEWVGNIKQDGSEKYYVDSVKGRFTISRDNAKNSLYLQMNSLRAEDTAVYYCARDLGVLWFG-DPYWGQGTLVTVSS</td></tr>
</table>

Table S1. Supplementary Table 1: Sequence alignments of RBD+ and Spike+ serum antibodies to public COVID sequences found in CoVAbDab. CDR-H3 sequences are delimited by a space.

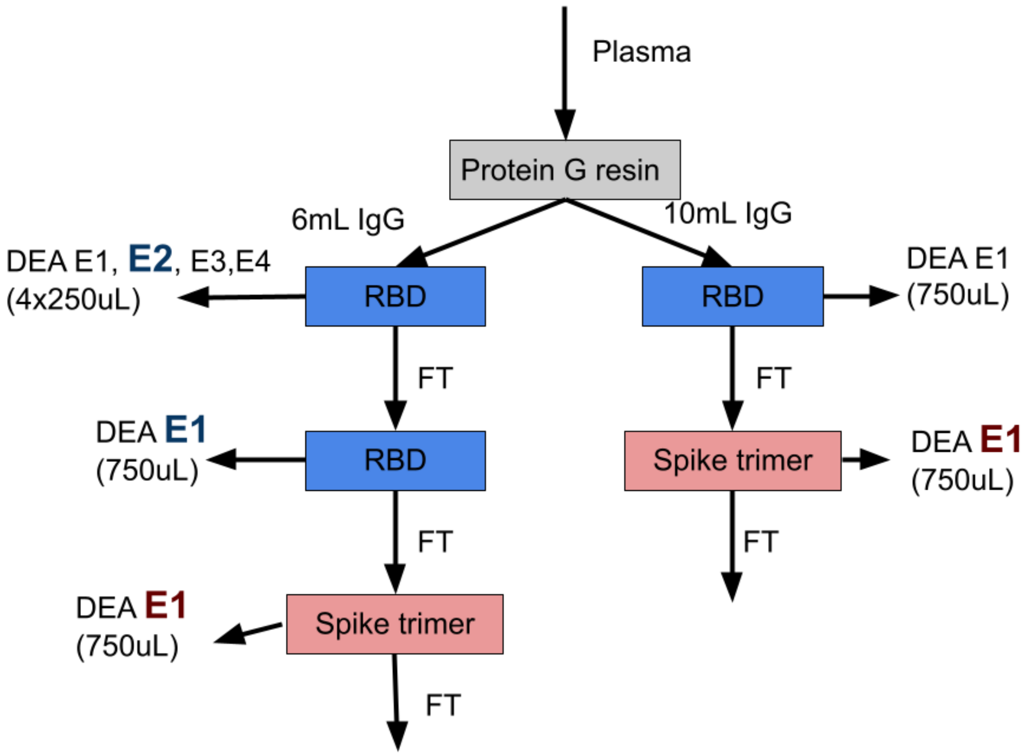

Figure S1. Supplementary Figure 1: Affinity purification strategy that was repeated for each donor plasma collections. Lines represent flow of plasma or antibodies, and boxes represent columns containing affinity resin: Protein G, RBD-conjugated, or Spike trimer-conjugated. Elution volumes in separate fractions are marked as E1, E2, E3, and E4. Elutions marked in bold were subjected to heavy and light chain separation via SDS-PAGE in reducing conditions, and four protease digestion to generate MS/MS data for each digestion, chain, elution, and donor. For Alicanto analysis, MS/MS data was aggregated to find anti-RBD antibodies (blue 6mL RBD+ and 6mL RBD+RBD+ elution runs) or anti-Spike antibodies (red 6mL Spike+ and 10mL Spike+ elution runs) per donor.
